# Examination of the relationship between essential genes in PPI network and hub proteins in reverse nearest neighbor topology

**DOI:** 10.1186/1471-2105-11-505

**Published:** 2010-10-12

**Authors:** Kang Ning, Hoong Kee Ng, Sriganesh Srihari, Hon Wai Leong, Alexey I Nesvizhskii

**Affiliations:** 1 Department of Pathology, University of Michigan, 4237 Medical Science Building I, Ann Arbor, MI, 48109, USA; 2 Center for Computational Biology and Medicine, University of Michigan, Ann Arbor, MI, 48109, USA; 3 Department of Computer Science, National University of Singapore, 117417, Singapore

## Abstract

**Background:**

In many protein-protein interaction (PPI) networks, densely connected hub proteins are more likely to be essential proteins. This is referred to as the "centrality-lethality rule", which indicates that the topological placement of a protein in PPI network is connected with its biological essentiality. Though such connections are observed in many PPI networks, the underlying topological properties for these connections are not yet clearly understood. Some suggested putative connections are the involvement of essential proteins in the maintenance of overall network connections, or that they play a role in essential protein clusters. In this work, we have attempted to examine the placement of essential proteins and the network topology from a different perspective by determining the correlation of protein essentiality and reverse nearest neighbor topology (RNN).

**Results:**

The RNN topology is a weighted directed graph derived from PPI network, and it is a natural representation of the topological dependences between proteins within the PPI network. Similar to the original PPI network, we have observed that essential proteins tend to be hub proteins in RNN topology. Additionally, essential genes are enriched in clusters containing many hub proteins in RNN topology (RNN protein clusters). Based on these two properties of essential genes in RNN topology, we have proposed a new measure; the *RNN cluster centrality*. Results from a variety of PPI networks demonstrate that *RNN cluster centrality *outperforms other centrality measures with regard to the proportion of selected proteins that are essential proteins. We also investigated the biological importance of RNN clusters.

**Conclusions:**

This study reveals that *RNN cluster centrality *provides the best correlation of protein essentiality and placement of proteins in PPI network. Additionally, merged RNN clusters were found to be topologically important in that essential proteins are significantly enriched in RNN clusters, and biologically important because they play an important role in many Gene Ontology (GO) processes.

## Background

Essential genes may cause the death of an organism if they are not properly expressed or malfunction due to events such as sequence mutation. Essential genes are vital for the growth of an organism under a variety of conditions and are frequently identified experimentally through deletion experiments (by the analysis of haploid deletion mutant strain growth rates)[1-3].

Recent high-throughput proteomic experiments, such as yeast-two hybrid [4] and affinity capture-MS [5,6], have enabled the systematic mapping of protein-protein interaction (PPI) for organisms such as *Saccharomyces cerevisiae *[4-6] and *Escherichia coli *[7]. Though the PPI networks constructed from these experiments are not yet complete, they nonetheless have revealed interesting topological properties of PPI networks [8,9] with respect to gene essentiality.

Specifically, several studies have already investigated the connection of the topological properties of PPI networks and essential genes [10-13]. The PPI network is represented as an unweighted, undirected graph, in which each node represents a protein and each edge (between two nodes) represents an interaction between these two proteins. In many PPI networks, essentiality is correlated with topological placement of the proteins in the network. That is, hubs that are "highly connected" in a PPI network tend to correspond to essential genes[10-17]. This is called the "centrality-lethality rule" [10]. Though the centrality-lethality rule has been observed in many PPI networks, the underlying topological properties of essential proteins are not yet fully understood. Jeong and colleagues argued that essential proteins are important in PPI network for maintaining the overall network connectivity, [10], while He and colleagues suggested that the majority of essential proteins are correlated with essential protein-protein interactions [11] in the PPI network. A recent study by Zotenko and colleagues utilizing a yeast PPI network, however, rejected these two suggestions, and proposed that proteins are essential due to their involvement in densely connected clusters of proteins with same GO term annotation [12]. Other works have shown high correlations between protein essentiality and their placement in protein complexes [18-20].

RNN topology was a weighted directed graph that could be generated from PPI network. In RNN topology, each of the nodes represented a protein, and each edge pointed to a protein from its RNN (with that protein as it nearest neighbor) [21,22]. RNN topology is different from nearest neighbor (NN) topology, since for each protein, its NN proteins and RNN proteins comprised two different protein sets. Since edges in RNN topology are both weighted and directed, they are useful for the identification of hub proteins that are important to the entire network. As with other topology modeling applications, the RNN topology can elucidate the topological, but not necessarily the true biological, dependencies between proteins. Nevertheless, there is an intricate correlation between topological dependencies and biological dependencies in PPI networks, as discussed in [10]. Therefore, the investigation of correlations between hub proteins in RNN topology and their essentiality could provide additional interesting insights such as whether these hub proteins play an important role in GO processes.

In this study, we explored the connection between topological properties of proteins and essential genes from a different perspective. Namely, we generated reverse nearest neighbor (RNN) topology [21,22] from the PPI network, and subsequently examined the connection of essential proteins and their placement in RNN topology, as well as the topological context in which essential proteins were enriched in RNN topology using different types of PPI networks. Our results show that essential proteins are more likely to be proteins with many RNNs. Additionally, essential proteins are enriched in clusters (RNN clusters) of proteins in RNN topology (referred to as the "clustering property" of essential proteins). Based on these observations, we propose the *RNN cluster centrality *measure, which is superior to other centrality measures in correlating hub proteins and essential proteins. Furthermore, we have observed that the RNN clusters play an important role in many GO processes.

## Methods

### Experimental data

The computational analysis was performed using PPI networks from two organisms, *E. Coli *K12 and budding yeast. For *E. Coli*, "DIP core" protein-protein interactions were retrieved from the DIP database [23] (http://dip.doe-mbi.ucla.edu/dip, accessed on 01/26/2009). The "DIP core" network was derived from "DIP full", with evolutionary information used to filter out unreliable interactions. Essential genes used in this study were identified based on a genome-wide targeted mutagenesis project [1] (http://ecogene.org, accessed on 07/01/2009).

The "DIP core" network for yeast was also obtained as described above. Several additional yeast PPI networks from different experiments were retrieved from BioGRID [24] (http://www.thebiogrid.org, version 2.0.52). These networks included two PPI networks generated by affinity capture-MS experiments: Krogan et al. [5] and Gavin et al. networks [25]; Collins et al. network [26], generated by the application of a statistical scoring scheme and filtering of low confidence score interactions from Krogan and Gavin's networks; and high confidence network (HC network) [27], which was generated by the intersection of small-scale datasets (including affinity capture-MS, yeast two-hybrid, etc.) with high throughput datasets [28]. The list of essential genes for yeast was obtained from *Saccharomyces *Genome Deletion Project [2,3] (http://www-sequence.stanford.edu/group/yeast_deletion_project/deletions3.html, accessed on 07/01/2009).

Known protein complexes were also used for comparison. The list of protein complexes was retrieved from a recent study of protein complexes in yeast [18] (http://dags.stanford.edu/Complex/reference.txt, accessed on 09/14/2009.)

### Methodologies of data analysis

#### RNN topology generation

RNN topology was a weighted directed graph generated from the PPI network. The directions of edges in RNN topology indicated that one node (edge destination) was another node's (edge origin) nearest neighbor, with the edge origin referred to as the RNN of the edge destination. The weights of edges indicated the topological importance of one protein to the other: that is, the lighter the edge weight, the closer (topologically) the edge origin is to the edge of destination.

**Figure 1:**
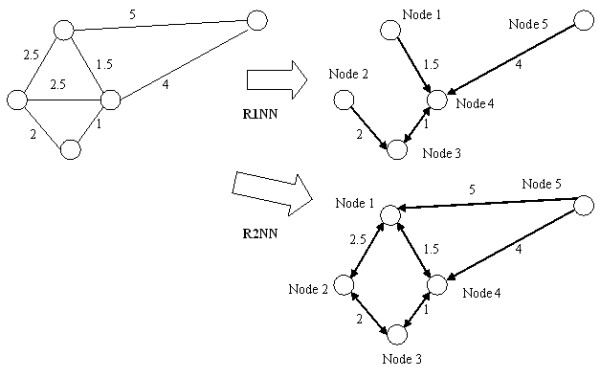
**An example of R1NN and R2NN topology generated from a toy weighted PPI network**. Numbers beside edges represent edge weights. The direction of the edges indicates that on node (edge destination) is another node (edge origin)'s nearest neighbor. The R1NN and R2NN from the PPI network were given.

RNN topology was generated from the original PPI network as follows. As the first step, the CMC algorithm [29] was applied to compute edge weights in the PPI network based on the connectivity of proteins in the PPI network. We have then transformed every edge weight in this weighted graph so that the closer the two proteins in RNN topology, the lower edge weight in the RNN topology. By this means, the more reliable the interaction, the closer the interaction in the RNN topology. Therefore, low edge weight in RNN topology after transformation actually indicates reliable interaction. Then RNN topology was generated based on this weighted PPI network by an efficient metric space search scheme [22] that identified the RNNs for every protein in the weighted PPI network. For each protein in RNN topology, other proteins with this protein among their top *k *nearest neighbors were referred to as the RkNN of this protein. The RkNN topology consisted of all proteins and the edges to every protein from their RkNNs. The generation of RNN topologies on a toy weighted PPI network is shown in Figure 1. For example, in Figure 1, Node 2 has no R1NN, and its R2NNs are Node 1 and Node 3. Note that some interactions in PPI network may not be present in RNN topology, since they represent weak dependencies of RNNs to the corresponding protein. It was found that there were special properties for RkNN with different *k*: Given any protein *q*, R1NN(q) is a subset of R2NN(q), R2NN(q) is a subset of R3NN(q), and so on. If *k *≥ *n-1 *(where n is the number of all proteins in the PPI network), then RkNN includes every interaction in the original PPI network. In real applications, it is of interest to analyze RkNN primarily for small *k *values.

#### RNN cluster generation

Based on RNN topology, we analyzed two types of clusters; simple and merged clusters. To generate simple clusters, all proteins in RNN topology were ranked by the number of their RNNs. The clusters were then generated by iteration. In each iteration, both the top-ranking protein and its RNNs (which were still present in the ranked list) formed a simple cluster, and all of these proteins were removed from the ranked list. This process was continued until there was no remaining protein in the ranked list.

For a cluster *I *in RNN topology, the *RNN cluster connectivity(I)*, was defined as the ratio of the number of RNN edges from proteins outside of RNN cluster to proteins inside the cluster, divided by the total number of edges pointing to proteins in this RNN cluster. The *RNN cluster connectivity *measure indicated the topological importance of RNN clusters (see RESULTS for discussion). Higher *RNN cluster connectivity *indicates higher connectivity of corresponding RNN cluster in the whole RNN topology. Therefore, the *RNN cluster connectivity *indicates connectivity of RNN clusters at the level of clusters of proteins rather than individual proteins. Using the *RNN cluster connectivity *measure, merged RNN clusters were then created by iteratively merging simple clusters and previously merged clusters. In each iteration, two simple (or merged) RNN clusters with the highest *RNN cluster connectivity *were merged if the resulting merged RNN cluster had a *RNN cluster connectivity *greater than a certain threshold. In this study, the threshold was set to be 0.5 for the balance between the quality and the number of merged clusters. This process was continued until there were no remaining proteins (clusters) that could be merged. All RNN clusters with *RNN cluster connectivity *smaller than 0.5 were subsequently filtered out.

#### Centrality measures for proteins

In this work, hub proteins were defined as proteins with high centrality values. Here, we introduce several centrality measures that are based either on RNN topology or the original PPI network. First, suppose the number of RNNs for each of the protein *i *is *RNN(i)*. The *RNN centrality *for a protein i is defined as the number of RNNs for this protein.

(1) RNN centrality (i)=RNN (i)

*RNN centrality *may be important for distinguishing essential and non-essential proteins. Note that this *RNN centrality *is also dependent on the types of RkNN used. For the same protein, when *k *value increases, the *RNN centrality *value also increases.

Second, we define *RNN cluster centrality*, which takes into consideration both *RNN centrality *and the enrichment of essential proteins in RNN clusters (clustering property). The enrichment of essential proteins in RNN clusters is represented by the *RNN cluster connectivity *measure described above. The *RNN cluster centrality *for each protein *i *in RNN cluster *I *is defined as

(2) RNN cluster centrality (i)=RNN (i)∗RNN cluster connectivity(I)|(i∈I)

For clustering methods other than RNN clustering, the clustering centralities are defined in a similar way. Suppose the number of interactions for protein *i *is degree(i) in the PPI network. The *cluster connectivity*(I) for cluster I is thus defined as the ratio of the number of interactions between one protein inside and another outside of this cluster, divided by the total number of interactions connecting proteins in the cluster. The *clustering centrality *is defined as:

(3) Clustering centrality (i)=degree(i)∗cluster connectivity(I)|(i∈I)

#### Measures for comparison

To compare the performance of different centrality measures in terms of their ability to identify essential proteins, two metrics are used. One metric is the "proportion of essential proteins in selected proteins" (*Precision*):

(4) Precision=#essential proteins selected/#proteins selected

The proteins are selected by their centrality measures (formula (1)-(3)). "# Essential proteins selected" is the number of proteins in the selected set of proteins. Note that the *precision *value is directly related to the centrality-lethality rule: the higher the proportion, the better the discrimination between essential and non-essential proteins provided by the centrality measure.

Another measure is the "proportion of essential proteins selected", *Recall*, which is the number of essential proteins selected in proportion to the total number of essential proteins in the dataset.

(5) Recall=#essential proteins selected/#all essential proteins

## Results and discussion

We have analyzed the connection between the placement of proteins in RNN topology and essential proteins based on different PPI networks. These include five networks for yeast: HC, Krogan, Gavin, Collins and DIP core, as well as DIP core network for *E. Coli *(see Methods). Among these PPI networks, the yeast HC network is presented here as a model network for most of the experimentation. Detailed statistics of these PPI networks are shown in Table 1.

**Table 1:** Statistics of the tested PPI networks.

Organism	PPI network name	Number of proteins	Number of interactions	Average degree	Number (Proportion) of essential proteins
**E Coli**	DIP Core	1,223	993	1.62	113 (0.09)

**Yeast**	DIP Core	3,645	4,553	2.50	690 (0.19)
	Krogan	2,674	7,079	5.29	712 (0.27)
	Gavin	1,430	7,592	10.62	576 (0.40)
	Collins	1,958	23,486	23.99	622 (0.31)
	HC	2,998	9,258	6.18	871 (0.29)

Proportion of essential proteins was computed as the number of essential proteins, divided by the total number of proteins in the PPI network.

The RNN topology is a scale free network [30], in which the distribution of the number of RNN connections follows a power law. In RNN topology, there are only a few proteins (hub proteins) that are the nearest neighbors for a large number of proteins (Additional file 1, Figure S1 and Additional file 1, Figure S2). These proteins are especially interesting since, having a large number (>6) of RNNs, there are more essential proteins than non-essential proteins (Additional file 1, Figure S2).

### Generation of RNN topology and assessment of RNN centrality measures

**Figure 2:**
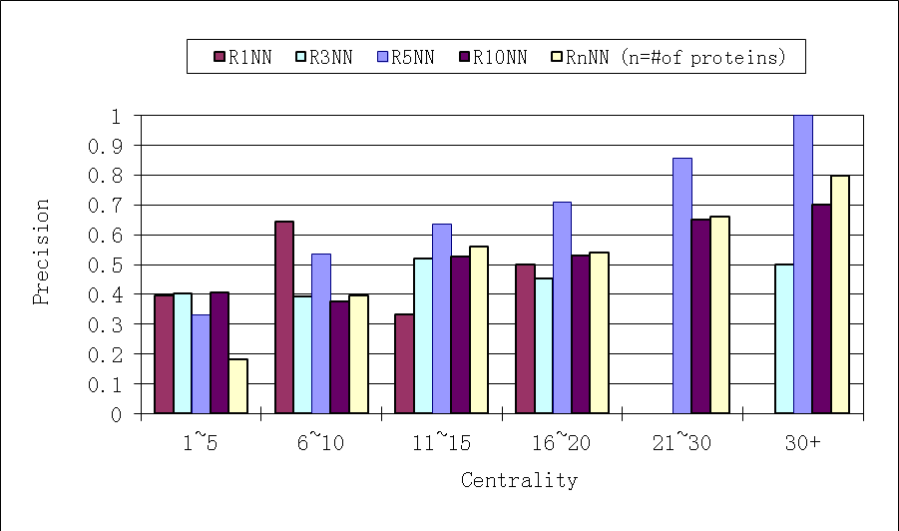
**The proportion of essential proteins (*precision*) (y-axis) in proteins categorized by values of centrality (x-axis)**. Results are based on RkNN with different *k *values generated from yeast HC PPI network. The higher the centrality values, the higher proportion of essential proteins could be observed.

The connection of *RNN centrality *and essential proteins was first analyzed based on the yeast HC PPI network. In the RkNN topology network, each protein has weighted edges pointing to it from other proteins, which in turn consider this protein to be among their top *k *nearest neighbors (see details in Methods). Based on RkNN topologies with different *k *values, it was observed that for RkNN with k > 5, increasing *precision *is observed in protein categories with increasing *RNN centrality*. However, this is not obvious for RkNN with smaller *k *values (see Figure 2). This may be due to a filtering effect: RkNN topologies with small *k *values may have filtered out so many edges from original PPI network, that the correlation of essential proteins and hub proteins could not be established. On the other hand, higher *precision *could be obtained from R5NN rather than from other RkNN (*k*>5) for protein categories with *RNN centrality *≥ 6 (Figure 2). The underlying reason is that RkNN with *k*>5 would include most edges in weighted PPI network, therefore rendering RNN topology less advantageous to the original PPI network. To determine this, we have computed the *RNN centrality *values based on RnNN (n = # of proteins), which would be exactly the same as degree centrality values (calculated as the degrees of proteins in the PPI network) computed on the original PPI network for every protein. We also observed that *RNN centrality *values based on R10NN are not significantly different from those based on RnNN (Figure 2). Therefore, the best *RNN centrality *values were obtained based on RkNN with *k *value not too large (>10) or too small (<5). Based on this analysis, we selected R5NN to represent RNN topology, and used it to perform all analyses described in the remainder of this manuscript.

#### Comparing RNN centrality with other centrality measures

**Figure 3:**
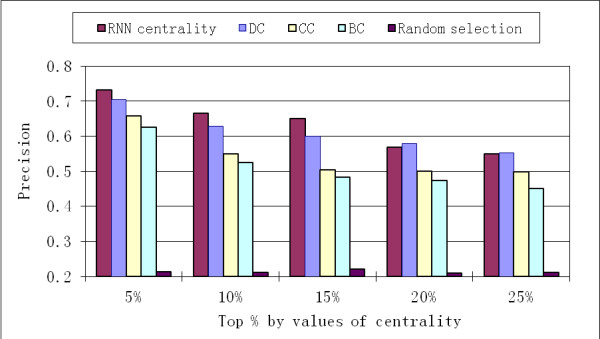
**The proportion of essential proteins (*precision*) in proteins ranked by different centrality measures**. *RNN centrality *is compared with DC (degree centrality), CC (closeness centrality), BC (betweenness centrality) and centrality based on random selection. Results were based on yeast HC PPI network. In each category, the top % of proteins with highest centrality values were computed by different methods and compared.

*RNN centrality *was compared with simple centrality measures based on the original PPI network. These centrality measures included the following: degree centrality (DC), which is calculated as the degree of the specific protein in the PPI network; closeness centrality (CC), which is calculated as the sum of the lengths of shortest paths from all other proteins to the specific protein in the PPI network; and betweenness centrality (BC) [16], which is the number of all-to-all shortest paths that go through the specific protein in the PPI network. A random selection method, based on the average result from ten runs of random sampling of proteins in PPI network, was also used for comparison of these centrality measures. Results show that all of these centrality measures were much better than random selection (Figure 3). Additionally, *RNN centrality *was superior to other centrality measures with regard to *precision *values.

### Assessment of RNN cluster centrality

**Figure 4:**
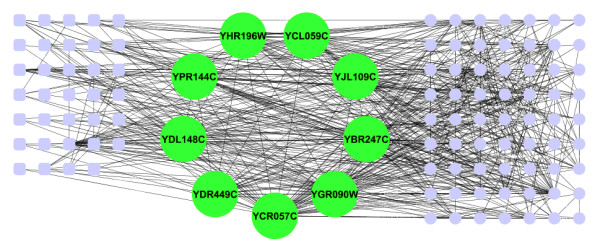
**A graphical representation of a sub-network of RNN topology derived from yeast HC PPI network**. Large green nodes represent a RNN cluster, small nodes represent the R1NNs of it members, circles represent essential proteins, and rectangle nodes (all in small nodes) represent non-essential proteins. All of the proteins in this RNN cluster are essential, and out of their 97 R1NNs, 63 (64.9%) are also essential proteins.

Since PPI networks are incomplete and noisy, it is not sufficient to distinguish essential and non-essential proteins based solely on a single protein's property, such as the *RNN centrality*. Alternatively, previous research has shown that essential proteins are more likely to be in densely connected clusters [31]. Here, we also analyzed the enrichment of essential proteins in RNN clusters (clustering property). Proteins with a higher proportion of their RNNs as essential proteins were more likely to be essential proteins, indicating that essential proteins tend to cluster together in RNN topology (see example Figure 4, also Additional file 1, Figure S3),. More specifically, in Figure 4, all of the 9 proteins in the RNN cluster were essential, and out of their 97 R1NNs, 63 (64.9%) were also essential proteins. This illustrates the importance of analyzing the connection of essential proteins and protein clusters (RNN clusters) in RNN topology.

First, we analyzed the enrichment of essential proteins in RNN clusters (see Methods for details of the clustering procedure). We compared the "proportion of essential proteins identified" (*recall*) values based on all merged RNN clusters (defined in Methods) with that based on random selection of the same number of proteins in PPI network. In the HC yeast PPI network, 87% of the essential proteins in the whole PPI network were within merged RNN clusters, as compared to 29% from random selection of proteins (Table 2). For other PPI networks, similar results were also observed: more than 70% of the essential proteins in the whole PPI network were within merged RNN clusters (*recall *= 70% ~ 90%), compared to only *recall *= 20~40% from random selection of proteins (Table 2).

**Table 2:** Comparison of "proportion of essential proteins identified" (*recall*) based on merged RNN cluster and that based on random selection of the same number of proteins in PPI network.

Organism	PPI network	*Recall *(merged RNN cluster)	*Recall *(random selection)	p-value
**E Coli**	DIP Core	0.89	0.09	0.0002

**Yeast**	DIP Core	0.73	0.19	0.0116
	Krogan	0.81	0.27	0.0183
	Gavin	0.85	0.40	0.0036
	Collins	0.79	0.31	0.0110
	HC	0.87	0.29	0.0014

Results were based on different PPI networks. p-value indicates the probability that the *recall *value is obtained based on random selection of proteins.

The topological importance of RNN clusters was examined by analyzing the effect of removing merged RNN clusters (including protein members and corresponding edges) on the change of topological properties of the PPI network. By comparing the average degree, betweenness, and closeness per protein in the original PPI network and those after removing all (100%) merged RNN clusters, we observed that these topological properties decreased to nearly half of their original values (Table 3). Furthermore, the removal of merged RNN cluster significantly deteriorated the network properties as compared to removal of the same number of randomly selected proteins (Table 3).

**Table 3:** The effect of removal of RNN clusters on the change of topological properties of yeast HC PPI network.

Remove top % RNN clusters	0%	10%	20%	50%	100%	p-value
**Average Degree per protein**	6.18	5.61 (5.94)	4.94 (5.83)	4.15 (5.39)	3.55 (4.79)	0.021

**Average Betweenness per protein**	0.190	0.073 (0.210)	0.0069 (0.207)	0.063 (0.196)	0.057 (0.192)	0.002

**Average Closeness per protein**	0.103	0.084 (0.088)	0.080 (0.084)	0.065 (0.081)	0.059 (0.074)	0.023

Values in brackets were based on random removal of the same number of proteins as in top % RNN clusters. p-values were associated with observing changes by random removal in PPI network.

#### Analysis of properties of RNN clusters

**Figure 5:**
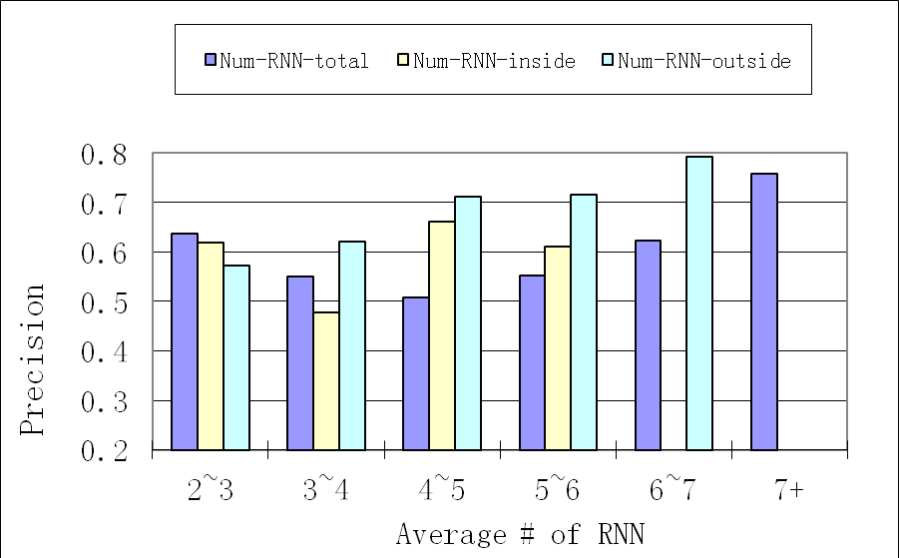
**The correlation of proportion of essential proteins (*precision*) and the average number of RNNs (of different types) per protein in merged RNN clusters**. Results are based on yeast HC PPI network. For the same proteins, the results are categorized by the number of total RNNs for this protein, the number of RNNs outside RNN clusters for this protein, and the number of RNNs inside RNN cluster for this protein.

Next, we performed the analysis of various properties of simple and merged clusters. Out of 1,677 proteins, there were 565 simple clusters with average size of 2.87, out of which more than 70% were of size less than 4. There were 309 merged clusters with average size of 5.01, out of which less than 30% were of size less than 4. Then we have analyzed the properties that could accurately represent the enrichment of essential proteins in RNN clusters (clustering property). We have examined three properties: (1) *Num-RNN-total *measured as the average number of edges pointing to a protein in RNN cluster; (2) *Num-RNN-inside *measured as the average number of edges pointing from one protein in RNN cluster to another protein in RNN cluster; and (3) *Num-RNN-outside *which was similarly defined. As shown in Figure 5, as the *Num-RNN-outside *increases, on merged RNN clusters, there is a corresponding linear increase of the *precision *value. However, both *Num-RNN-total *and *Num-RNN-inside *do not have such a linear correlation with *precision*. The same phenomenon was observed when the basis was simple RNN clusters (details not shown). Therefore, among these three properties, *Num-RNN-outside *reflects the enrichment of essential proteins in RNN cluster. This is also the underlying principal defining *RNN cluster connectivity *(refer to METHODS section).

Additionally, the cluster density on merged RNN clusters was also analyzed. The cluster density is defined as the number of edges in a cluster, divided by the number of all possible edges between proteins in the cluster. However, it was discovered that increasing the threshold of density of RNN clusters did not result in increased *precision *in RNN clusters (see Additional file 1, Figure S4 (a)). This indicated that cluster density was not good at discriminating between essential and non-essential proteins.

**Figure 6:**
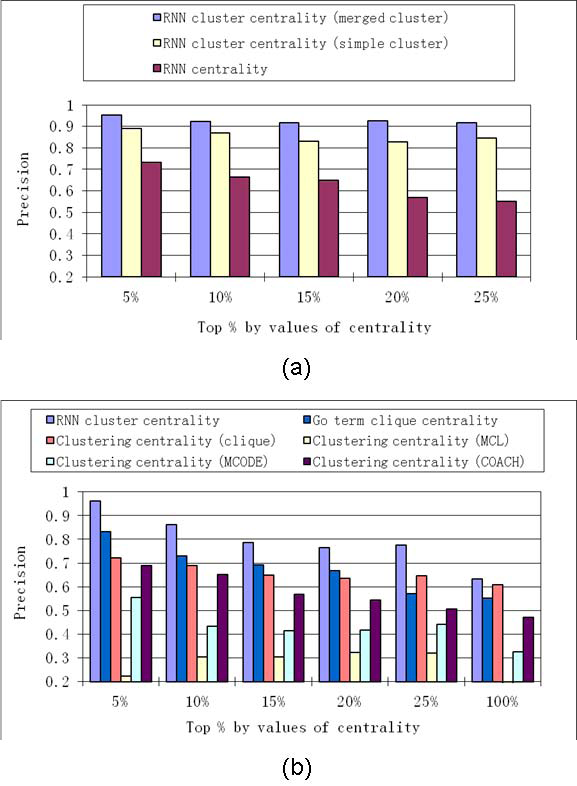
**Comparison of RNN cluster centrality with other centrality measures in proteins ranked by different centrality measures**. (a) Shows the comparison of *precision *based on *RNN cluster centrality *with *precision *based on *RNN centrality*. (b) Shows the comparison of *precision *by *RNN cluster centrality *with *precision *based on other centrality measures based on clustering methods. Results are based on yeast HC PPI network.

The simple RNN clusters and merged RNN clusters were compared by computing the *RNN cluster centrality *based on single RNN clusters, with those that based on merged RNN clusters. The *RNN cluster centrality *based on merged RNN clusters as consistently better than that based on simple RNN clusters, with regard to *precision *values (Figure 6 (a)s). For simplicity, hereafter, "*RNN cluster centrality*" is referred to that computed for a merged RNN cluster.

#### Comparison of RNN cluster centrality with other centralities

By comparing *RNN cluster centrality *with *RNN centrality*, *precision *values were consistently much higher when based on *RNN cluster centrality *rather than *RNN centrality *(see Figure 6 (a)). This indicated that essential proteins were more likely to be hub proteins inside RNN clusters than hub proteins outside of the RNN cluster.

*RNN cluster centrality *was then compared with *clustering centrality *measures based on several clustering methods applied to PPI networks. The clustering methods that we compared included the following: MCODE, which is based on network density [32]; the MCL method, based on random walk [33]; the COACH method, based on core-attachment structure [34]; and clustering by cliques [31]. A clique in PPI network is a subgraph in which each of the proteins is connected with all other proteins in the same subgraph. Recently, a research study was conducted on clustering proteins in PPI network based on GO function annotation [12], in which densely connected proteins with same GO term annotations were considered to be in the same cluster. Since essential proteins tend to be enriched in cliques [31] and in clusters with the same GO term annotations [12], we introduced the "GO term cliques" where essential proteins were expected to be highly enriched. The "GO term cliques" were created as follows: proteins with the same GO function annotation [35] from the PPI network were clustered, and then cliques (with number of proteins > 2) from these clusters were extracted as GO term cliques. Note that "GO term cliques" was a stringent term, since all proteins in the clique should have the same GO function annotation and also connect to all other proteins in the same clique. Formula (3) was used to compute clustering centralities based on these clustering methods.

Results on the yeast HC PPI network show that *RNN cluster centrality *had superior *precision *values relative to other clustering centralities (Figure 6 (b)). Among all of these centrality measures, the *RNN cluster centrality *was more effectively discriminated between essential and non-essential proteins. It is worth noting that the superior results of *RNN centrality *were obtained without utilizing functional annotation information as is the case for GO term clique. Additionally, though similarly high *precision *values could be obtained from GO term clique centrality (Figure 6 (b)), higher *recall *values were obtained from all RNN clusters than from those obtained from GO term cliques. Based on the yeast HC PPI network, 754 essential proteins (out of all 871 essential proteins, *recall *= 0.87) were identified from all RNN clusters, while only 172 essential proteins (*recall *= 0.20) were identified from all GO term cliques. The main reason for low *recall *values based on GO term cliques is that GO term cliques contained only a small fraction of proteins in the PPI network; out of all 2,998 proteins in yeast HC PPI network, only 311 were members of GO term cliques. On the other hand, 1,192 proteins in the yeast HC PPI network were members of merged RNN clusters.

We also compared different cluster centrality measures on other yeast PPI networks, as well as an *E.Coli *PPI network. Results from these PPI networks indicated that *RNN cluster centrality *and GO term clique centrality gave consistently higher *precision *values (Additional file 1, Figure S5). Additionally, in the *E. Coli *PPI network, *RNN cluster centrality *yielded superior *precision *values as compared to other centrality measures (Additional file 1, Figure S5). This also indicated that *RNN cluster centrality *was superior for examination of correlations of network topology with essential proteins, and this was independent of the organisms on which the PPI network was established.

### Assessment of biological importance of RNN cluster centrality

#### Comparison of merged RNN clusters with known protein complexes

Previous studies [20] suggest that proteins were essential due to their involvement in densely connected and biologically meaningful clusters of proteins, such as protein clusters sharing the same GO term annotation [12] and protein complexes [20]. As we have related here, merged RNN clusters were comparable to GO term cliques with regard to the enrichment of essential proteins. Here, we compared merged RNN clusters with known protein complexes [18].

Results based on the yeast HC PPI network show that there were significant overlaps between RNN clusters and protein complexes: out of the 309 merged RNN clusters, 115 (37.2%) had higher than 20% protein overlaps (computed as the # of overlapping proteins/# proteins in complex) with 126 (29.8%) out of 423 reference protein complexes, and 81 (26.2%) with least 50% protein overlaps with 81 (19.1%) reference protein complexes. The amount of overlap was high, which is even comparable to the results of some of the most recent work on protein complex prediction [36].

**Figure 7:**
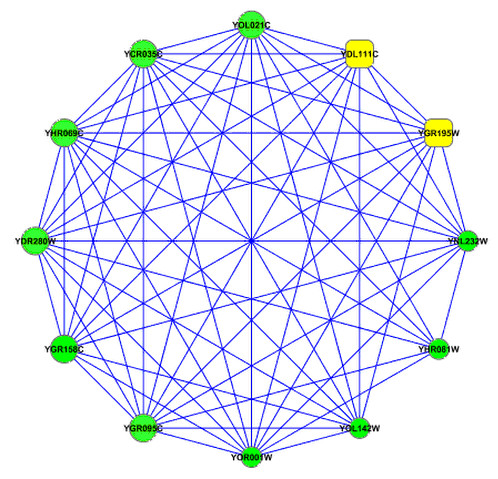
**A graphical representation of the sub-network in yeast HC PPI network**. All nodes belong to a protein complex (SG_0000176). Large nodes represent members of a RNN cluster, small nodes represent non-members of this RNN cluster, circles represent essential proteins and rectangle nodes represent non-essential proteins.

An example of a merged RNN cluster in a yeast HC PPI network consisted of 8 proteins, all these 8 proteins belonged to a protein complex for rRNA processing that consisted of 12 proteins (complex SG_0000176 from SGD [37]) (Figure 7). All of these 8 proteins and their connections composed of a big clique, while adding the other 4 proteins outside of the merged RNN cluster would result in a non-clique. This indicated that proteins are very densely connected. From these 8 proteins, 6 were found to be essential proteins, as identified by gene deletion experiments [2]. The other two proteins (YDL111C for gene RRP42 and YGR195W for gene SKI6) were also essential (refer to http://www.yeastgenome.org), but they were not identified by the gene deletion experiments. Additionally, except for protein YGR095C, 7 out of 8 proteins in this RNN cluster are exosome complex exonuclease proteins. While in the other 4 proteins that are members of protein complex SG_0000176 but not members of this RNN cluster, only two proteins are exosome complex exonuclease proteins.

**Figure 8:**
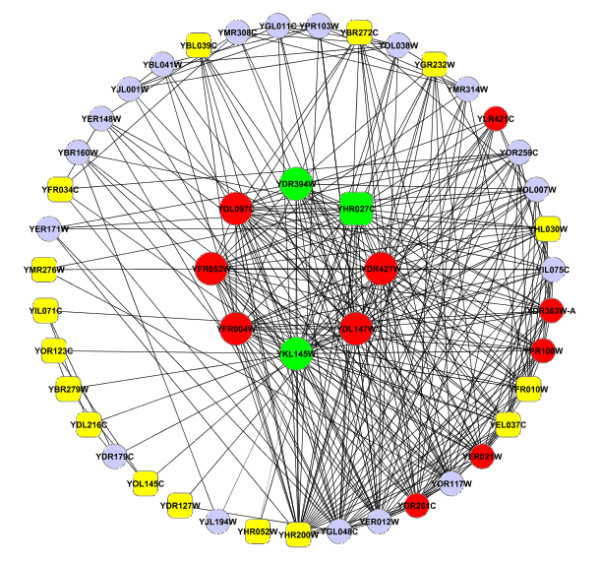
**A graphical representation of the sub-network in yeast HC PPI network**. Large nodes (inner circle) represent members of a RNN cluster; small nodes (outer circle) represent R5NN of proteins in the RNN cluster. Circles represent essential proteins and rectangle nodes represent non-essential proteins. Nodes in red represent all 10 members of a protein complex (SG_0008541).

Another example of merged RNN cluster in a yeast HC PPI network consisted of 8 proteins (Figure 8). All of these 8 proteins were in the 26S proteasome regulatory particle chain. Out of these 8 proteins, only one was not an essential proteins, and its degree in R5NN topology was ranked 5th out of 8 proteins. Out of these 8 proteins, 5 belonged to a protein complex (SG_0008541). This protein complex contained 10 members, and the other 5 members of this protein complex were within the R5NN for proteins in this RNN cluster. The proportion of essential proteins within the R5NN was 59.0% (23/39), which was lower than the enrichment of essential proteins in RNN cluster 87.5% (7/8) but higher than the PPI network average (29%). This indicated that (1) essential proteins tended to be enriched in R5NN topology and (2) essential proteins were more enriched in RNN clusters. Additionally, we have noticed that among all proteins in this RNN cluster and their R5NN, the members of the RNN cluster did not have high connectivity, indicating that density-based methods may not work well on clustering essential proteins.

From "DIP core" *E. Coli *PPI network, a merged RNN cluster consisted of 5 proteins: DIP-9704N (FtsL), DIP-9703N (FtsK), DIP-9706N (FtsQ), DIP-9702N (PBP-3) and DIP-12117N (YgbQ). Except for the last one, which was a hypothetical protein, 4 proteins in this merged RNN cluster were essential proteins. The protein DIP-9704N, DIP-9703N, DIP-9706N and DIP-12117N were all cell division protein, and DIP-12117N was also a Penicillin-binding protein. Together, DIP-9704N, DIP-9703N and DIP-9706N could be members of different protein complexes, though sometimes DIP-9704N, DIP-9706N could both be members of a complex without DIP-9703N [38]. As respect to structure, the localization of DIP-9704N was dependent on DIP-9703N and DIP-9706N, and DIP-9702N's localization required DIP-9704N and DIP-9703N [39].

Comparing RNN clusters with protein complexes, it was also observed that the enrichment of essential proteins is more significant in merged RNN clusters than in protein complexes; we have defined *protein complex centrality *in the same way as *clustering centrality*, and discovered that *RNN cluster centrality *is superior to protein complex centrality with regard to *precision *values (Additional file 1, Figure S6).

#### Analysis of the connection between merged RNN clusters and GO processes

The significant overlap between merged RNN clusters and known protein complexes suggests that merged RNN clusters have some biological importance. To further investigate this possibility, we analyzed the connection between merged RNN clusters and GO processes. We extracted all GO processes that contain at least one merged RNN cluster (referred to as "important GO processes" in [12]), and generated GO sub-networks so that each of these sub-networks contains proteins of the same GO process. Following these operations, the proportion of essential proteins in these GO sub-networks was computed. It was apparent that the enrichment of essential proteins in proteins that are in GO sub-network and merged RNN clusters was usually higher than the enrichment of essential proteins in GO sub-networks, which indicates that merged RNN clusters play an important role in these GO processes (Table 4). In GO processes that are highly enriched in essential proteins, such as RNA metabolic process (GO:0016070), the essential proteins were also distributed unevenly (with regard to inside and outside of the RNN clusters): For RNA metabolic process, 69% of proteins in GO sub-network were essential proteins, while for proteins that are both in the GO sub-network and in the merged RNN cluster, 85% are essential proteins. This property of merged RNN clusters is similar to that of essential complex biological modules (ECOBIMs) that are introduced in [12], suggesting that there is a low tolerance of merged RNN clusters to biological perturbation. However, it is worth noting that biological annotation, such as GO, was not used in creating the RNN clusters, whereas ECOBIMs were generated based on such information. From the above results, it was suggested that both the placement of the protein in the RNN topology and the GO process annotation of the protein are very good predictors of protein essentiality.

**Table 4:** The enrichment of essential proteins in merged RNN clusters for GO sub-networks in the yeast HC PPI network.

GO term	Name	Proportion of essential proteins in		
		**GO sub-network**	**GO sub-network and merged RNN clusters**	**GO sub-network but not in merged RNN clusters**

GO:0042254	ribosome biogenesis	0.69	0.85	0.63
GO:0016070	RNA metabolic process	0.48	0.66	0.41
GO:0006997	nucleus organization	0.47	0.60	0.44
GO:0007059	chromosome segregation	0.47	0.50	0.46
GO:0070271	protein complex biogenesis	0.44	0.67	0.39
GO:0007049	cell cycle	0.36	0.48	0.34
GO:0044257	cellular protein catabolic process	0.36	0.81	0.21
GO:0006350	transcription	0.35	0.50	0.30
GO:0006259	DNA metabolic process	0.33	0.44	0.30
GO:0006457	protein folding	0.33	0.13	0.36
GO:0051276	chromosome organization	0.30	0.37	0.28
GO:0006810	transport	0.29	0.52	0.26
GO:0016192	vesicle-mediated transport	0.27	0.55	0.22
GO:0006412	translation	0.25	0.47	0.21
GO:0016044	membrane organization	0.24	0.46	0.21
GO:0006464	protein modification process	0.20	0.38	0.16
GO:0006950	response to stress	0.20	0.39	0.16
GO:0000746	conjugation	0.19	0.20	0.18
GO:0007126	meiosis	0.17	0.35	0.14
GO:0044262	cellular carbohydrate metabolic process	0.17	0.57	0.14
GO:0007165	signal transduction	0.15	0.30	0.13
GO:0019725	cellular homeostasis	0.08	0.08	0.08
GO:0042221	response to chemical stimulus	0.07	0.17	0.06

Each of the GO sub-networks contains at least one merged RNN cluster; the proportion of essential proteins in "proteins in GO sub-network", "proteins in GO sub-network and also in merged RNN clusters", and "proteins in GO sub-network but not in merged RNN clusters".

#### Analysis of putative types of hubs

Some debate has persisted in the literature regarding the possible distinction between "date" and "party" hubs ([40]) in the PPI network. In this work, we have also tried to analyze whether any significant difference is detectable between the two putative hub types using RNN cluster centrality measure. The date/party distinction is a biologically meaningful property, and we have used the intersection of hub proteins derived from our work and those from [27]. Based on yeast HC PPI network, we deleted either putative date hubs or putative party hubs in descending order of RNN cluster centrality from the PPI network, and computed the average closeness of the proteins of the remaining part in the PPI network. It was observed that there was not much difference between the deletion of putative date hubs and the deletion of putative party hubs: when 50% of putative date hubs were deleted, the average closeness was 0.076, while deletion of 50% of putative party hubs resulted in the average closeness of 0.073. Therefore, there was not much topological difference between date and party hubs in the PPI network as regard to hub deletion from PPI network based on RNN cluster centrality.

## Conclusions

In this work, we have examined the placement of essential proteins in RNN topology. The RNN topology is a weighted directed graph generated from PPI network, in which the topological dependencies of one protein to the others are elucidated. Based on different types of PPI networks, we found that proteins with many RNNs (high *RNN centrality *values) are more likely to be essential proteins. Additionally, it was observed that essential proteins tend to be enriched in RNN clusters (i.e., clustering property of essential proteins). This finding was consistent with recent reports, suggesting that essential proteins tend to be members of densely connected clusters [20]. Moreover, we have shown that RNN clusters have a higher proportion of essential proteins than other types of clusters. We have also introduced the *RNN cluster essentiality*. And demonstrated that it was constantly superior to *RNN centrality *and other clustering centrality measures, e.g., *clustering centrality *based on cliques, with regard to the proportion of selected proteins that are essential proteins. Furthermore, we have analyzed the connection between merged RNN clusters and GO processes, and discovered that enrichment of essential proteins in the intersection of a GO sub-network and merged RNN clusters is generally higher than the enrichment of essential proteins in GO sub-networks alone. This indicated that the placement of the protein in the RNN topology and the GO process annotation of the protein are both important predictors of protein essentiality. Therefore, future work should include a meta centrality measurement, such as UniScore [13] based on several existing methods, that combines both the *RNN cluster centrality *and the GO term for increased power to discriminate between essential and non-essential proteins.

## Authors' contributions

KN conceived the study, proposed the methods, performed the experiments, wrote and revised the manuscript. HKN and SS performed the experiments. HWL and AIN revised the manuscript. All authors have read and approved this manuscript.

## Appendix

KN's current address: Qingdao Institute of BioEnergy and Bioprocess Technology, Chinese Academy of Sciences. Qingdao, Shandong, China. Email: ningkang@qibebt.ac.cn.

## Supplementary Material

Additional file 1 **Additional figures for the paper**. Figure S1. The weighted yeast HC PPI network. Figure S2. The distribution of the frequency (number) of essential and non-essential proteins. Figure S3. Number of proteins and proportion of essential proteins in these proteins (y-axis) categorized by the proportion of essential proteins as their RNN (x-axis). Figure S4. The effect of cluster density on essential protein identification in RNN topology. Figure S5. The proportion of essential proteins in top proteins on different PPI networks. Figure S6. The proportion of essential proteins in top proteins ranked by different centrality measures. Click here for file
