# Supplementary material for: Examination of the relationship between essential genes in PPI network and hub proteins in reverse nearest neighbor topology
Source: BMC Bioinformatics. 2010 Oct 12;11:505. doi: 10.1186/1471-2105-11-505 (PMC3098085; doi:10.1186/1471-2105-11-505)
Supplement: Additional file 1 — Additional figures for the paper. Figure S1. The weighted yeast HC PPI network. Figure S2. The distribution of the frequency (number) of essential and non-essential proteins. Figure S3. Number of proteins and proportion of essential proteins in these proteins (y-axis) categorized by the proportion of essential proteins as their RNN (x-axis). Figure S4. The effect of cluster density on essential protein identification in RNN topology. Figure S5. The proportion of essential proteins in top proteins on different PPI networks. Figure S6. The proportion of essential proteins in top proteins ranked by different centrality measures. [file 1471-2105-11-505-S1.DOC]

# Additional Files: Examination of the relationship between essential genes in PPI network and hub proteins in reverse nearest neighbor topology

### Kang Ning1[[1]](#footnote-2)*, Hoong Kee Ng^3^, Sriganesh Srihari^3^, Hon Wai Leong^3^ and Alexey I. Nesvizhskii^1,2,§^

^1^Department of Pathology, University of Michigan, 4237 Medical Science Building I, Ann Arbor, MI, USA, 48109

^2^Center for Computational Biology and Medicine, University of Michigan, Ann Arbor, MI, USA, 48109

^3^Department of Computer Science, National University of Singapore, Singapore 117417

^§^Corresponding author

Email addresses:

KN: [kning@](mailto:kning@)umich.edu

HKN: nghoongk@comp.nus.edu.sg

SS: srigsri@comp.nus.edu.sg

HWL: leonghw@comp.nus.edu.sg

AIN: [nesvi@umich.edu](mailto:nesvi@med.umich.edu)


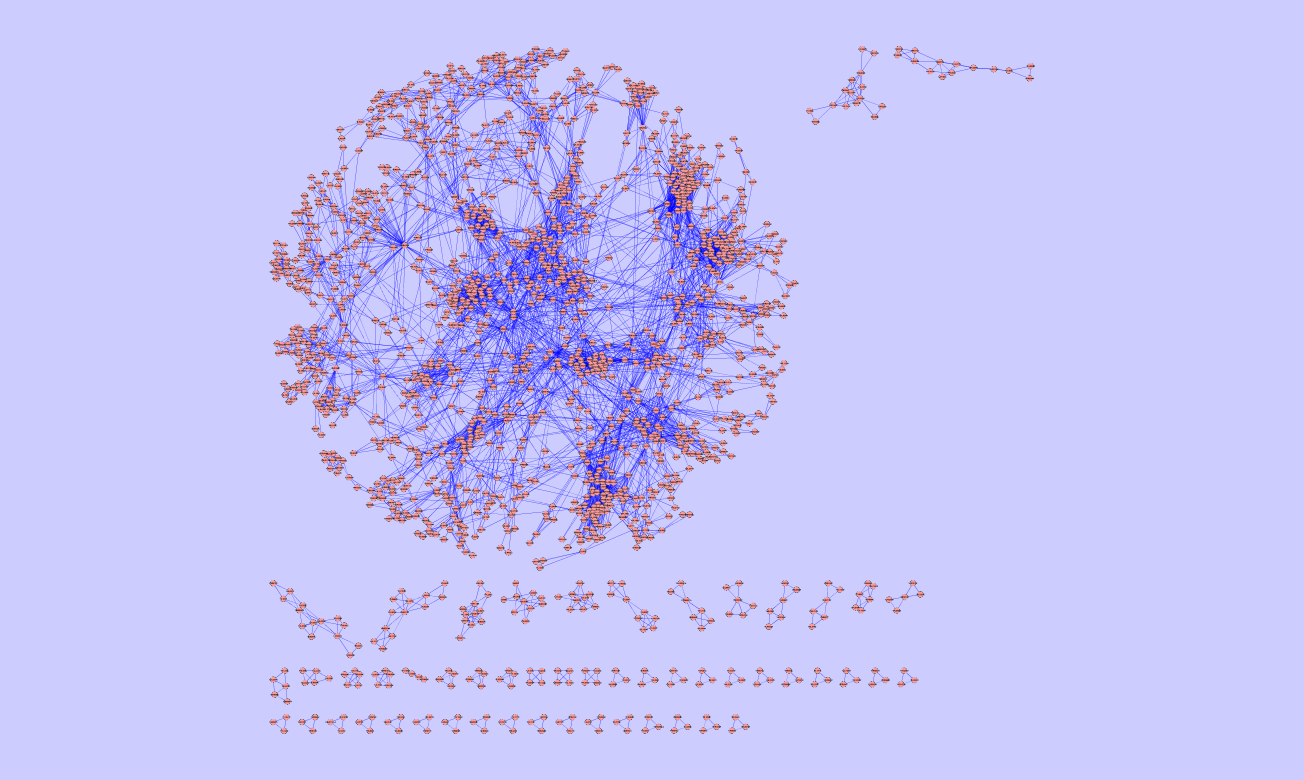


Figure S1. The weighted yeast HC PPI network.

Figure S2. The distribution of the frequency (number) of essential and non-essential proteins categorized by the number of their RNNs. Results were based on yeast HC PPI network.

|  |
| --- |
| (a) |
|  |
| (b) |

Figure S3. Number of proteins and proportion of essential proteins in these proteins (y-axis) categorized by the proportion of essential proteins as their RNN (x-axis). (a) is based on R1NN and (b) is based R5NN and R5NN without proteins of RNN < 5. Resultswere based on yeast HC PPI network.


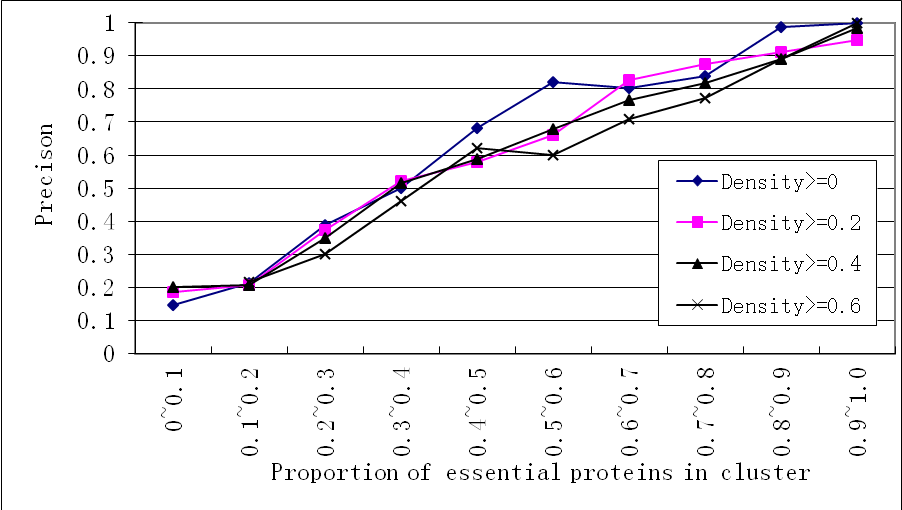


Figure S4. The effect of cluster density on essential protein identification in RNN topology. Results show *precision* against the proportion of essential proteins in clusters. *Precision* is computed based on proteins with *RNN centrality* > 10 in RNN cluster. Results were based on merged RNN clusters from yeast HC PPI network.

| 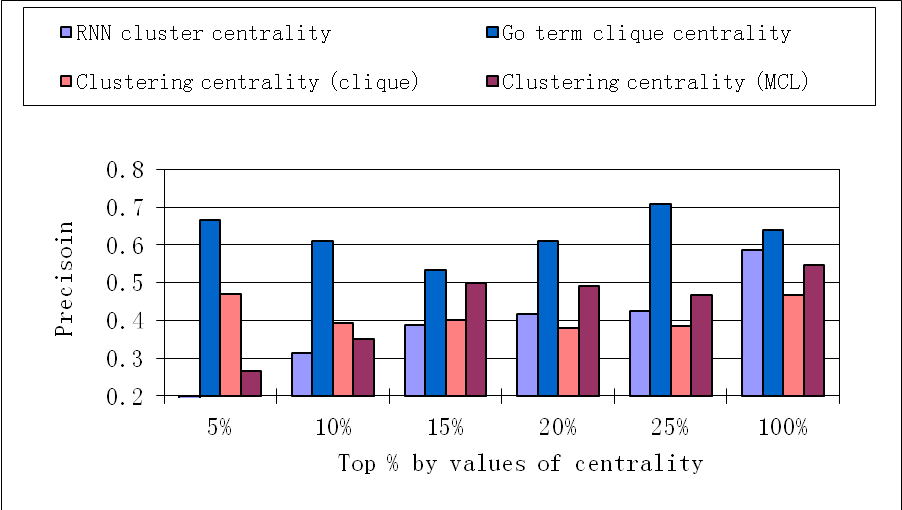 |
| --- |
| (a) Krogan |
| 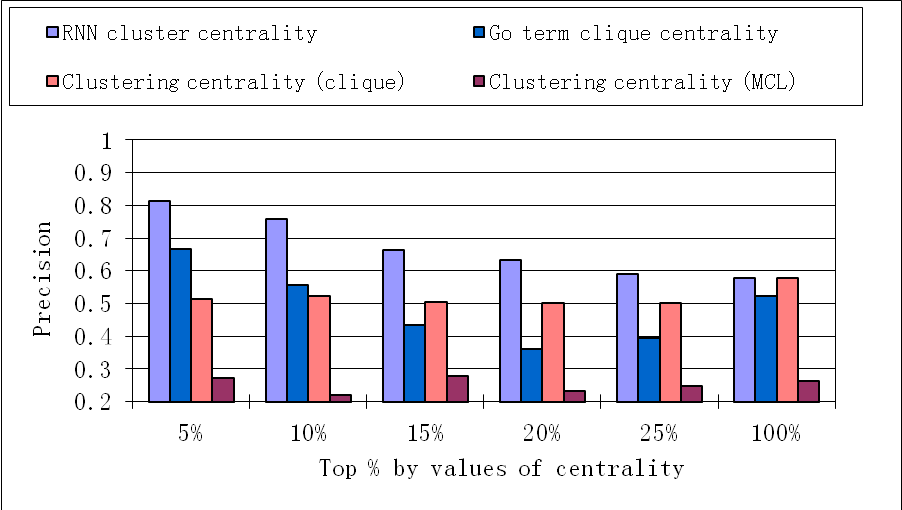 |
| (b) Gavin |
| 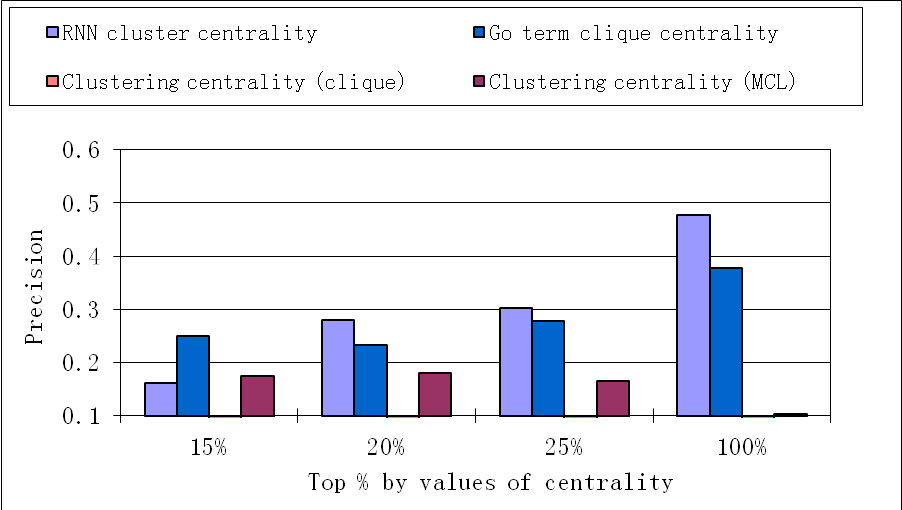 |
| (c) Collins |
| **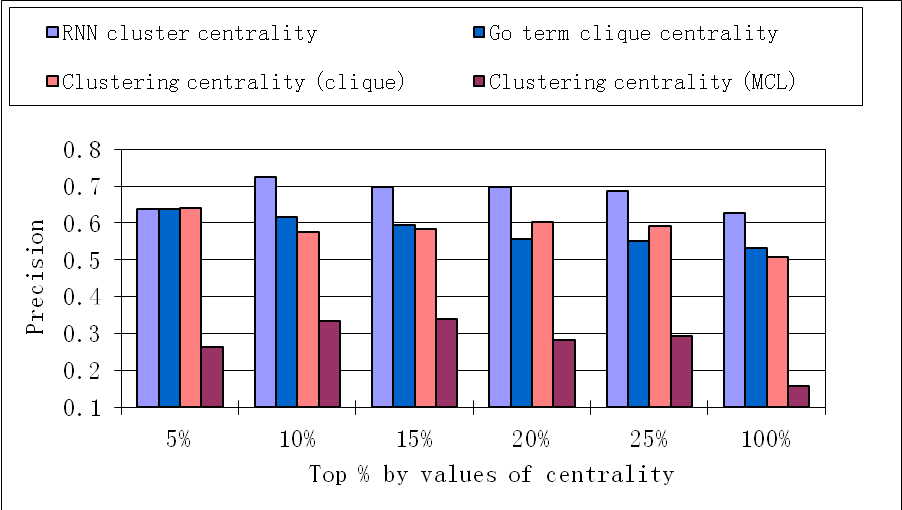** |
| (d) DIP Core for yeast |
| 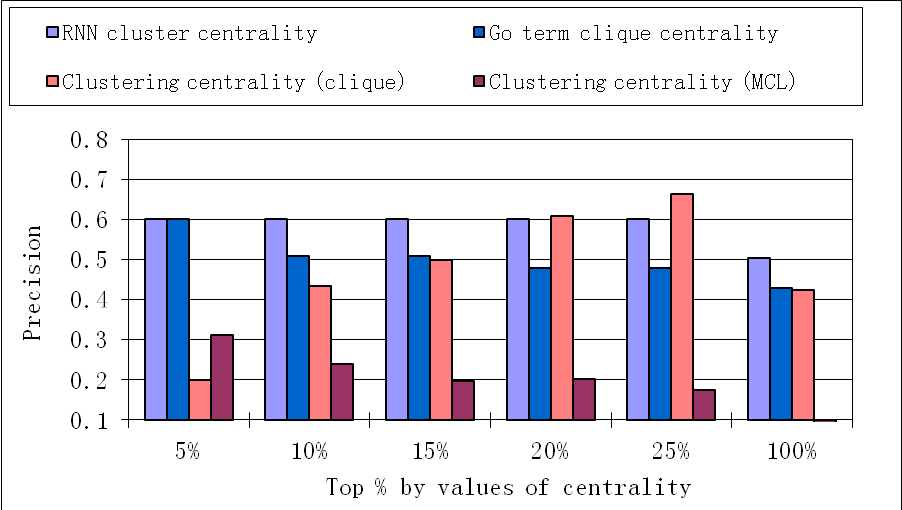 |
| (e) DIP Core for E. Coli |

Figure S5. The proportion of essential proteins in top proteins on different PPI networks. For Collins PPI network, there were few clusters by GO term centrality measure, therefore results are shown starting from top 15% of clusters ranked by value of centrality.

| 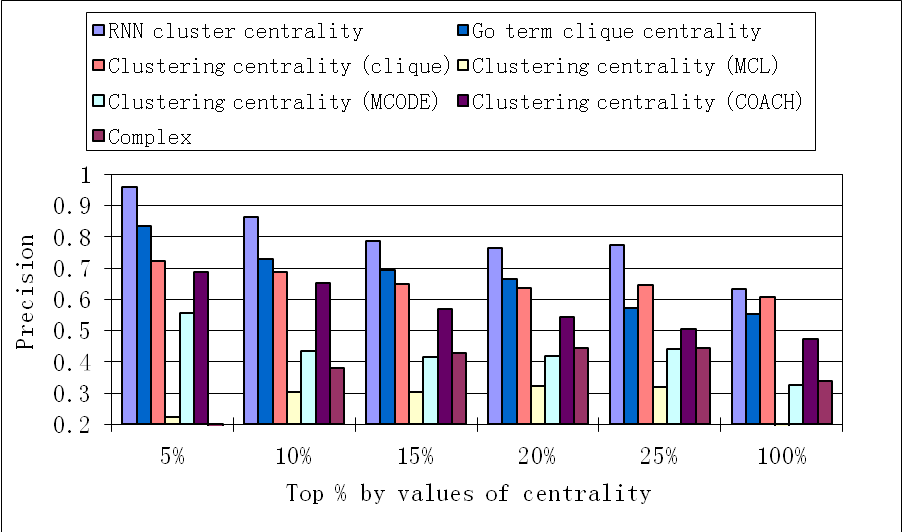 |
| --- |

Figure S6. The proportion of essential proteins in top proteins ranked by different centrality measures. Results were based on yeast HC PPI network.

1. ^*^ Current Address: Qingdao Institute of BioEnergy and Bioprocess Technology, Chinese Academy of Sciences. Qingdao, Shandong, China. Email: ningkang@qibebt.ac.cn. [↑](#footnote-ref-2)
